# Prediction of disease-free survival for precision medicine using cooperative learning on multi-omic data

**DOI:** 10.1093/bib/bbae267

**Published:** 2024-06-05

**Authors:** Georg Hahn, Dmitry Prokopenko, Julian Hecker, Sharon M Lutz, Kristina Mullin, Leinal Sejour, Winston Hide, Ioannis Vlachos, Stacia DeSantis, Rudolph E Tanzi, Christoph Lange

**Affiliations:** Department of Biostatistics, Harvard T.H. Chan School of Public Health, 677 Huntington Ave, 02115, Boston, MA, USA; Department of Neurology, Genetics and Aging Research Unit, McCance Center for Brain Health, Massachusetts General Hospital, 55 Fruit Street, 02114, Boston, MA, USA; Channing Divsion of Network Medicine, Brigham and Women’s Hospital and Harvard Medical School, 75 Francis Street, 02115, Boston, MA, USA; Department of Biostatistics, Harvard T.H. Chan School of Public Health, 677 Huntington Ave, 02115, Boston, MA, USA; Department of Neurology, Genetics and Aging Research Unit, McCance Center for Brain Health, Massachusetts General Hospital, 55 Fruit Street, 02114, Boston, MA, USA; Department of Pathology, Beth Israel Deaconess Medical Center, 330 Brookline Avenue, 02215, Boston, MA, USA; Department of Pathology, Beth Israel Deaconess Medical Center, 330 Brookline Avenue, 02215, Boston, MA, USA; Department of Pathology, Beth Israel Deaconess Medical Center, 330 Brookline Avenue, 02215, Boston, MA, USA; Houston Campus, The University of Texas Health Science Center, 1200 Pressler Street, 77030, Houston, TX, USA; Department of Neurology, Genetics and Aging Research Unit, McCance Center for Brain Health, Massachusetts General Hospital, 55 Fruit Street, 02114, Boston, MA, USA; Department of Biostatistics, Harvard T.H. Chan School of Public Health, 677 Huntington Ave, 02115, Boston, MA, USA

**Keywords:** Alzheimer, cooperative learning, Cox proportional hazard, lasso, penalized regression, precision medicine, survival

## Abstract

In precision medicine, both predicting the disease susceptibility of an individual and forecasting its disease-free survival are areas of key research. Besides the classical epidemiological predictor variables, data from multiple (omic) platforms are increasingly available. To integrate this wealth of information, we propose new methodology to combine both cooperative learning, a recent approach to leverage the predictive power of several datasets, and polygenic hazard score models. Polygenic hazard score models provide a practitioner with a more differentiated view of the predicted disease-free survival than the one given by merely a point estimate, for instance computed with a polygenic risk score. Our aim is to leverage the advantages of cooperative learning for the computation of polygenic hazard score models via Cox’s proportional hazard model, thereby improving the prediction of the disease-free survival. In our experimental study, we apply our methodology to forecast the disease-free survival for Alzheimer’s disease (AD) using three layers of data. One layer contains epidemiological variables such as sex, APOE (apolipoprotein E, a genetic risk factor for AD) status and 10 leading principal components. Another layer contains selected genomic loci, and the last layer contains methylation data for selected CpG sites. We demonstrate that the survival curves computed via cooperative learning yield an AUC of around $0.7$, above the state-of-the-art performance of its competitors. Importantly, the proposed methodology returns (1) a linear score that can be easily interpreted (in contrast to machine learning approaches), and (2) a weighting of the predictive power of the involved data layers, allowing for an assessment of the importance of each omic (or other) platform. Similarly to polygenic hazard score models, our methodology also allows one to compute individual survival curves for each patient.

## Introduction

In the age of precision medicine, both predicting the susceptibility of an individual to a certain disease and forecasting its disease-free survival are areas of fundamental research. Many common diseases are complex and result from a combination of multiple genetic and environmental factors. Examples of such complex diseases include diabetes [1], obesity [2], schizophrenia [3, 4] or autism [5]. At the same time, the increasing availability of technologies, such as exome and genome sequencing, enables their use in clinical research and practice. They can thereby help to inform better treatments of complex diseases and, more importantly, the creation of (individual) predictive models of disease risk. These properties are crucial to efficiently allocate medical resources, thus ensuring both appropriate individual care and reducing the burdens on the healthcare system. However, it is usually not the case that a single technology is able to fully elucidate the complexity of a disease, apart from possibly some rare examples [6]. Therefore, we ideally seek to combine different technologies which provide useful views into both the phenotypes and the processes leading to the disease under investigation. This combination of different technologies leads to new statistical and interpretational challenges. Indeed, it is not straightforward to combine independently created datasets in order to draw valid (statistical) inference. In our case, we focus on the computation of polygenic hazard score models which provide a practitioner with a more differentiated view of the predicted disease-free survival than the one given by merely a point estimate, thus allowing a practitioner to better gauge the absolute risk of an individual as a function of time.

In this work, we present novel methodology which uses a recent statistical technique called *cooperative learning* to combine the predictive power of several data layers (that is, several data sources on the same cohort) for the computation of the disease-free survival using polygenic hazard score models, thereby improving the accuracy of the prediction. In contrast to machine learning approaches, the proposed methodology returns (1) a linear weighting of the predictors that can be easily interpreted, and (2) a weighting of the predictive power of the involved data layers, thus allowing for an assessment of the importance of each omic (or other) platform.

Polygenic hazard score models are computed via Cox’s proportional hazards [7, 8] and allow one to predict the time-dependent hazard and survival for a given individual under the assumption that censoring and event times are independent [9, 10]. Such time-dependent hazard and survival curves for an individual allow one to better predict the disease-free survival. They improve upon two shortcomings of the classic polygenic risk scores [11, 12], which are a common measure of the aggregated genetic risk for a disease or trait [13]. First, they provide an absolute risk measure, as opposed to the relative risk with respect to a reference population provided by a polygenic risk score. Second, they capture the time-dependent risk of an individual, whereas polygenic risk scores merely provide a point estimate.

Cooperative learning is a new framework to draw simultaneous inference from multiple datasets [14]. Improved technologies in biomedicine allow one to collect data from an increasing number of sources, for instance genomics, epigenomics, proteomics or metabolomics, which might all carry information about an outcome of interest. Combining the predictive power of those covariates has the potential to increase the accuracy of predictions or to make discoveries possible in the first place [6, 15]. However, fitting a model separately on each dataset usually results in predictions which do not agree. Cooperative learning is a novel approach to combine the predictive power of several datasets using the lasso [16] in connection with additional penalties to enforce consistency of the predictions across different datasets.

The presented methodology is very general in that it allows one to forecast the disease-free survival for an arbitrary disease or trait from an arbitrary number of datasets. We merely require that for the disease or trait of interest, $m \in{\mathbb{N}}$ datasets are given on the same number of $n \in{\mathbb{N}}$ individuals, where each dataset can contain an arbitrary number of predictors. For training the model, apart from the predictive datasets, the age of the last follow-up (for controls) or the age of onset (for cases) is required per individual. The fitted polygenic hazard score model then allows one to forecast the time-dependent hazard and survival for an unseen individual (for instance, in a validation set).

We showcase the proposed methodology using an example involving the prediction of the disease-free survival of Alzheimer’s disease (AD). The term *Alzheimer’s free survival* refers to the time during which an individual has not yet contracted AD. Three data layers are used to forecast the AD-free survival, each containing information for the same $457$ individuals ($236$ cases and $221$ controls). The first dataset contains $12$ epidemiological variables, precisely sex, APOE (apolipoprotein E, a genetic risk factor for AD) status and 10 leading principal components. The second dataset contains $83$ selected genomic loci from [17]. The third dataset contains methylation data of $201$ selected CpG (5’-C-phosphate-G-3’) sites for all individuals [18]. The CpG sites and variants were selected to be associated with AD. We demonstrate that in the case of AD-free survival prediction, the usage of cooperative learning to combine the predictive power of all three datasets yields survival curves with a state-of-the-art AUC while having the advantage of providing interpretable models (in contrast to machine learning approaches applied to the same three data layers). As already shown in [10], our methodology likewise allows one to compute individual survival curves for a patient, thus providing a practitioner with a more differentiated view of the absolute hazard or survival as a function of time.

The article is structured as follows. Section Methods revisits the methodology of polygenic hazard score models and cooperative learning, and demonstrates how both can be combined to base the computation of hazard and survival curves upon several data layers. Section Experimental results applies the methodology to the three datasets of epidemiological, genomic and methylation data for AD-free survival. The article concludes with a discussion in Section Discussion.

## Methods

We first revisit the polygenic hazard score models introduced in [10], which are based on a Cox’s proportional hazards model in connection with the lasso to incorporate additional covariates (Section Polygenic hazard score models). After revisiting cooperative learning in Section Incorporation of several data layers viacooperative learning, we introduce our methodology to combine polygenic hazard score models with cooperative learning to draw conclusions from multiple data layers (Section Cooperative polygenic hazard score models). The section closes with some computational considerations (Section Computationalconsiderations) and the computation of survival curves from hazard curves (Section Numerical integration of the hazard curve toobtain the survival curve).

### Polygenic hazard score models

We first consider one dataset $Z \in R^{n \times p}$ containing $p \in{\mathbb{N}}$ covariates for each of $n \in{\mathbb{N}}$ individuals. We denote each row in $Z$ with $Z^{(i)} \in{\mathbb{R}}^{p}$, where $i \in \{1,\ldots ,n\}$. For each individual $i \in \{1,\ldots ,n\}$, we aim to fit a Cox’s proportional hazards model [7, 8] augmented with the covariates $Z^{(i)} \in{\mathbb{R}}^{p}$, given by the parametric functional 


(1) \begin{align*} h(t|Z^{(i)}) = h_{0}(t) \exp \left( \beta^{\top} Z^{(i)} \right).\end{align*}

In eq. (1), the quantity $h_{0}(t)$ is a time dependent baseline hazard to be fitted separately on the data. The parameter $\beta \in{\mathbb{R}}^{p}$ is an unknown regression parameter which is fitted using the entire population of the $n$ individuals. We call eq. (1) a polygenic hazard score model.

Several methods are available to fit $\beta $. We follow the approach of [10] and employ the two-step procedure of [19], which is based on a partial log likelihood and allows for the inclusion of additional covariates into the Cox’s proportional hazards model. To be precise, let $T_{i}$ and $C_{i}$ be the failure time and the censoring time of individual $i \in \{1,\ldots ,n\}$, respectively. The partial log likelihood defined in [19] is given by 


(2) \begin{align*} l_{Z}(\beta) = \sum_{i=1}^{n} \delta_{i} \left[ \beta^{\top} Z^{(i)} - \log \left( \sum_{j=1}^{n} {\mathbb{I}}(T_{j} \geq T_{i}) \exp \left( \beta^{\top} Z^{(j)} \right) \right) \right],\end{align*}

where $\delta _{i} = {\mathbb{I}}(T_{i} \leq C_{i})$ is an indicator which encodes whether censoring of individual $i$ has taken place. Any individual having a failure time $T_{i}$ less or equal to the censoring time $C_{i}$ contributes to the partial log likelihood. Each individual contributes a regular regression term $\beta ^{\top } Z^{(i)} \in{\mathbb{R}}$ in eq. (2), normalized by the contributions of the other individuals $j$ with failure times $T_{j} \geq T_{i}$. The normalization becomes a substraction in eq. (2) after applying logarithms.

Instead of maximizing the partial log likelihood of eq. (2) directly for $\beta $, in [19] the authors add a standard lasso-type $L_{1}$ penalty for $\beta $, leading to the objective function 


(3) \begin{align*} \min_{\beta} \left[ -\frac{1}{n} l_{Z}(\beta) + \lambda |\beta/\hat{\beta}| \right],\end{align*}

where $\beta /\hat{\beta }$ denotes the componentwise division of vector entries and $|\cdot |$ denotes the $L_{1}$ norm. As can be seen, eq. (3) requires the maximizer vector $\hat{\beta }$ of the partial log likelihood of eq. (2), which has to be computed first and is then added to the penalty term in eq. (3) to ensure the consistency of the minimizer $\beta $, see [19]. The entries of $\hat{\beta }$ essentially act as a weighting in the lasso penalty. This is justified by the fact that $\hat{\beta }$ is a consistent estimator, thus its entries reflect the importance of the covariates [19]. As usual, the lasso parameter $\lambda $ in eq. (3) controls the level of sparsity. We will determine $\lambda $ with the help of cross validation.

The approach of using eq. (1) to compute individual polygenic hazard score models, where the vector $\beta $ is fitted with the help of eq. (3), is the *Cox-lasso* model of [10].

### Incorporation of several data layers via cooperative learning

In the previous section, eq. (2) and eq. (3) allow one to fit the parameter $\beta $ of the Cox’s proportional hazards model of eq. (1) using additional covariates. Those additional covariates are given in the matrix $Z \in{\mathbb{R}}^{n \times p}$, containing $p \in{\mathbb{N}}$ covariates for each of $n \in{\mathbb{N}}$ individuals.

We aim to extend the polygenic hazard score model of Section Polygenic hazard score models to multiple datasets using cooperative learning [14]. Cooperative learning addresses the scenario in which we are given $m \in{\mathbb{N}}$ datasets $X_{i} \in{\mathbb{R}}^{n \times p_{i}}$ for $i \in \{1,\ldots ,m\}$ that might all have predictive power for some outcome $y \in{\mathbb{R}}^{n}$ of interest. Each dataset contains information for all $n$ individuals. Naturally, the number of covariates in each dataset can differ, and it is thus denoted as $p_{i} \in{\mathbb{N}}$ for $i \in \{1,\ldots ,m\}$. Importantly, cooperative learning is not restricted to linear regression, but merely assumes that predictions of $y$ are computed with an arbitrary function $f_{i}$ on the dataset $X_{i}$ for each $i \in \{1,\ldots ,m\}$. Each function $f_{i}$ may depend on $d_{i} \in{\mathbb{N}}$ parameters, denoted as $\beta _{i} \in{\mathbb{R}}^{d_{i}}$. A straightforward choice is to set $f_{i}$ as a linear model, meaning $f_{i}(X) = X_{i} \beta _{i}$.

As outlined in [14], two extreme cases are straightforward. The first case is called *early fusion*, and it refers to the case in which all $m$ datasets are merged into one dataset $X \in{\mathbb{R}}^{n \times p}$ by matrix concatenation, where $p = \sum _{i=1}^{m} p_{i}$. We then predict $y$ from $X$ using a method of choice (encoded in a function $f$). The second case is called *late fusion*, and it refers to carrying out $m$ separate predictions $f_{1}(X_{1}),\ldots ,f_{m}(X_{m})$. Naturally, the resulting predictions differ, and thus they have to be fused into one coherent prediction of $y$. This can be done in a multitude of ways, with averaging all predictions being only one of many options.

Cooperative learning provides a framework to carry out the estimation and to enforce consistency among the estimates. Among other ways, this can be achieved (see eq. (20) in [14]) using the objective function 


(4) \begin{align*} \min_{\beta_{1},\ldots,\beta_{m},\rho} \left[ \frac{1}{2} \left( y - \sum_{i=1}^{m} f_{i}(X_{i}) \right)^{2} + \frac{\rho}{2} \sum_{i<j} \left( f_{i}(X_{i}) - f_{j}(X_{j}) \right)^{2} \right].\end{align*}

In eq. (4), each prediction function $f_{i}$ depends on the unknowns $\beta _{i}$ for $i \in \{1,\ldots ,m\}$, and the *agreement parameter*$\rho>0$ controls the weight with which the predictions based on the $m$ datasets are enforced to agree. The agreement parameter $\rho $ can either be chosen by the user, or fitted to the data. As $\rho \rightarrow 0$, solving eq. (4) tends toward performing late fusion, whereas $\rho \rightarrow \infty $ causes eq. (4) to enforce the prediction to agree at the expense of the prediction quality. 

### Cooperative polygenic hazard score models

We now state an adapted version of the polygenic hazard score model of Section Polygenic hazard score models. The adapted version allows one to fit a polygenic hazard score model to given failure times $T_{i}$ and censoring times $C_{i}$, where $i \in \{1,\ldots ,n\}$ and $n \in{\mathbb{N}}$ is the number of individuals, in the scenario that we are given $m \in{\mathbb{N}}$ data layers $Z_{1},\ldots ,Z_{m}$. Each dataset has dimensions $Z_{i} \in{\mathbb{R}}^{n \times p_{i}}$ with $p_{i} \in{\mathbb{N}}$, where $i \in \{1,\ldots ,m\}$.

Adapting eq. (1) to $m$ data layers leads to the cooperative polygenic hazard score model given by 


(5) \begin{align*} h(t|Z_{1}^{(i)},\ldots,Z_{m}^{(i)}) = h_{0}(t) \exp \left( \sum_{j=1}^{m} \beta_{j}^{\top} Z_{j}^{(i)} \right)\end{align*}

for individual $i \in \{1,\ldots ,n\}$. In eq. (5), $\beta _{j}$ are the fitted parameters for dataset $Z_{j}$ for all $j \in \{1,\ldots ,m\}$, and $h_{0}(t)$ is the time-dependent baseline hazard as in Section Polygenic hazard score models.

In order to fit all parameter vectors $\beta _{j}$, where $j \in \{1,\ldots ,m\}$, we leave the partial log likelihood of eq. (2) unchanged and adapt the lasso objective of eq. (3) with the help of cooperative learning in order to ensure the consistency of the estimates $\beta _{j}$, resulting in the minimization task 


(6) \begin{align*} \min_{\beta_{1},\ldots,\beta_{m}} \left[ -\frac{1}{n} \sum_{i=1}^{m} l_{Z_{i}}(\beta_{i}) + \frac{\rho}{2} \sum_{i<j} \left( Z_{i} \beta_{i} - Z_{j} \beta_{j} \right)^{2} + \lambda \sum_{j=1}^{m} |\beta_{j}/\hat{\beta}_{j}| \right].\end{align*}

In eq. (6), $\hat{\beta }_{j}$ are the fitted parameters obtained by maximizing the partial log likelihood $l_{Z_{j}}$ separately for $j \in \{1,\ldots ,m\}$. As in eq. (4), the parameter $\rho $ in eq. (6) controls the weight with which the consistency of the estimations based on the different datasets is enforced, and $\lambda $ is the lasso regularization parameter.

The model given in eq. (5), with parameters $\beta _{j}$ fitted to each dataset $Z_{j}$ for $j \in \{1,\ldots ,m\}$ using eq. (6), is the main contribution of this article. Together, both equations allow one to both compute individual polygenic hazard score models while drawing inference from several data layers simultaneously using cooperative learning.

**Figure 1:**
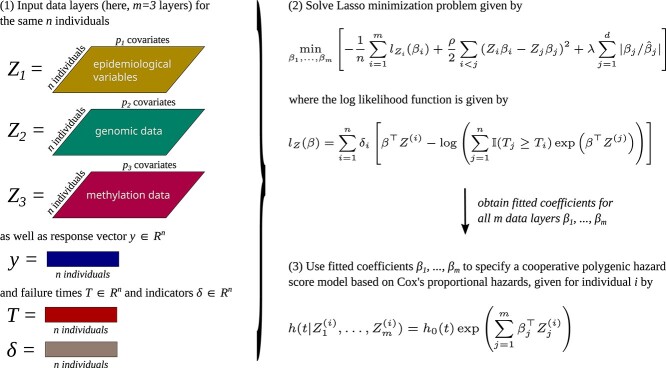
Flowchart of the methodology presented in Section Cooperative polygenic hazard score models. The $m \in{\mathbb{N}}$ input layers contain different data views on the same $n \in{\mathbb{N}}$ individuals, each with an independent number of covariates $p_{i} \in{\mathbb{N}}$ for $i \in \{1,\ldots ,m\}$. Additionally, we are given a response $y \in{\mathbb{R}}^{n}$, failure times $T \in{\mathbb{R}}^{n}$ and censoring indicators $\delta \in{\mathbb{R}}^{n}$ for all $n$ individuals (see Section Polygenic hazard score models), depicted in step (1). The model parameters $\beta _{1},\ldots ,\beta _{m}$ are fitted using a lasso-type minimization, see step (2). This yields $m$ fitted coefficients $\beta _{1} \in{\mathbb{R}}^{p_{1}},\ldots ,\beta _{m} \in{\mathbb{R}}^{p_{m}}$, one for each data layer. The fitted coefficients are then used to fully specify the cooperative polygenic hazard score model based on Cox’s proportional hazards for each individual $i \in \{1,\ldots ,n\}$, depicted in step (3). The framework can be encapsulated in a cross-validation step whereby only a part of the data of step (1) is being used for the fit.

A flowchart depicting the fitting process and final specification of the cooperative polygenic hazard score model based on Cox’s proportional hazards can be found in Figure 1.

### Computational considerations

Given a set of $m \in{\mathbb{N}}$ data layers $Z_{1},\ldots ,Z_{m}$ for $n \in{\mathbb{N}}$ individuals, the $m$ parameter vectors $\beta _{1},\ldots ,\beta _{m}$ are fitted by minimizing eq. (6). As discussed in Section Polygenic hazard score models, the required estimates $\hat{\beta }_{j}$ in eq. (6) are obtained first by maximizing the partial log likelihood of eq. (2) separately for each $Z_{j}$, where $j \in \{1,\ldots ,m\}$. We carry out all optimizations with the help of the *optim* function in R using the *BFGS* algorithm with numerical gradients. For added numerical stability, all estimates $\beta _{i}$ and $\hat{\beta }_{i}$ are normed to have an $L_{2}$ norm of one after optimization.

Once the parameter vector $\beta _{j}$ is obtained for each dataset $Z_{j}$, the cooperative polygenic hazard score model of eq. (5) is fully defined for each individual $i \in \{1,\ldots ,n\}$. After the optimization step is complete, the fitted parameters (on the training set) are kept fixed. For any unseen individual in a validation set, a fully specified cooperative polygenic hazard model is given by eq. (5) with all $\beta _{j}$ and the baseline hazard $h_{0}$ being fixed. To fit the baseline hazard, we employ the function *basehaz* of the *survival* package on CRAN [20].

In all experiments of Section Experimental results, we fit a cooperative polygenic hazard score model to a training set in the same way as described above, and evaluate the model on unseen individuals in a validation set.

### Numerical integration of the hazard curve to obtain the survival curve

Once eq. (5) is calibrated, it fully specifies the cooperative polygenic hazard score model on $m$ data layers. However, in Section Experimental results we also require the corresponding survival functions. Using the relation $h(t) = -\frac{d}{dt} \log S(t)$ between the hazard $h(t)$ and the survival $S(t)$, we compute the survival function for any individual $i \in \{1,\ldots ,n\}$ as 


(7) \begin{align*} S(t|Z_{1}^{(i)},\ldots,Z_{m}^{(i)}) = \exp \left( -\int_{0}^{t} h(x|Z_{1}^{(i)},\ldots,Z_{m}^{(i)}) dx \right)\end{align*}

via numerical integration.

## Experimental results

In this section, we present numerical results for the proposed polygenic hazard score model of eq. (5). Two types of datasets are used. First, we start with an introduction of three real data layers which we employ for prediction (Section Realdatasets on AD), followed by an overview of the competitive methods we include in our experiments (Section Experimentalsetup). Section Hazard and survival curves showcases the hazard and survival curves we obtain when fitting a cooperative polygenic hazard score model, and Section Comparisonwith other popular approaches compares our method to other popular approaches with respect to accuracy and runtime. Second, we consider simulated data consisting of genetic and methylation data layers (Section Simulated genetic and methylationdatasets), and use them to repeat our assessment of the cooperative polygenic hazard score model and its competitive methods. We conclude with an ablation study in Section Ablation study to assess the influence of the two simulated data layers (simulated genetic and methylation data).

### Real datasets on AD

We have used three layers of data from MGB Biobank (formerly Partners Biobank, see [21]). MGB Biobank is a hospital-based cohort from a collection of patient samples and information recruited at clinics throughout Mass General Brigham (MGB) hospitals [22]. The three data layers contain information on $457$ individuals, composed of $236$ AD (ICD-10) cases and $221$ controls.

Controls were selected to be matched by age, sex and APOE status (genotyped). The first dataset contained $12$ epidemiological variables, precisely sex, APOE (apolipoprotein E) status and the first 10 principal components which summarize the genomic data. The second dataset contains $83$ AD-associated genomic loci taken from [17] (genotypes were available through MGB Biobank). The third dataset contains normalized methylation levels for $201$ CpG sites for all individuals [18].

**Figure 2:**
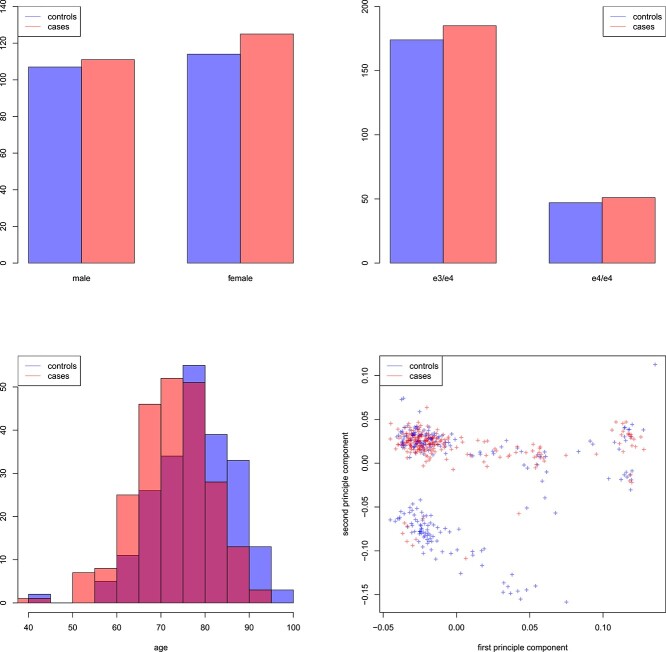
Selected characteristics of the real dataset of Section Real datasets on AD. Controls are depicted in blue, and cases are depicted in red. Histogram of the sex distribution (top left), the APOE status (top right), the age distribution (bottom left) and the first two principle components (bottom right).

Originally, methylation was performed on $464$ subjects, matched by age, sex and APOE status (genotyped). Blood methylation measurements were generated using an EPIC Methyl850k array on five plates from Illumina using the MGB PM Biobank Genomics Core. In the initial quality control we removed probes based on the following criteria: (1) $90\,374$ probes which contained SNPs that were identified in [23]; (2) $44\,735$ probes which had a detection *P*-value $<0.01$; (3) $15\,819$ probes which mapped to sex chromosomes; (4) $2626$ probes which mapped to CG start sites; (5) $2852$ probes which had a beadcount of less than 3. Additional quality control was performed with the R-package *ewastools* [24, 25]. We have excluded three samples which had a high odds of belonging to the outlier component across all SNP probes, as recommended in the *ewastools* vignette. Finally, in order to reduce possible noise and the number of predictors, we selected $201$ CpG sites which overlapped with the $220$ CpG sites shown to be associated with neuropathology [18]. A graphical summary of selected characteristics of the three data layers we use is given in Figure 2.

### Experimental setup

This section introduces both the metrics we employ for evaluation, as well as the other methods which we include in the comparisons of this article:

The approach of [9] is a popular model to predict the hazard and survival with the help of a forward stepwise Cox regression. The method itself is based on a conditional partial likelihood, and it is designed for one data source only. Using the notation of Section Methods, in [9] the authors define
  (8) \begin{align*} L(\beta) = \prod_{i=1}^{n} \frac{\exp(\beta^{\top} Z^{(i)})}{\sum_{k \in R(T_{i})} \exp(\beta^{\top} Z^{(k)})},\end{align*}
  where $R(T_{i}) = \{ j \in \{1,\ldots ,n\}: T_{j} \leq T_{i} \}$ is the set of other individuals $j$ whose event times $T_{j}$ are less or equal than $T_{i}$. After optimizing eq. (8) for $\beta $, which is done using the *optim* function in R with method *BFGS* and numerical gradients, the vector $\beta $ is normalized in $L_{2}$ norm as done in Section Computational considerations. The final hazard score model for [9] is likewise given by eq. (1), where the baseline hazard is estimated as done in Section Computational considerations.
The *Cox-lasso* of [10] is the predecessor of the presented algorithm. As introduced in Section Polygenic hazard scoremodels, it likewise fits a polygenic hazard score model to a single data source. To determine the fitted coefficients $\beta $, the Cox-lasso minimizes eq. (3). After normalization of the $\beta $ parameters, the final hazard score model is, as usual, given by eq. (1) with the baseline hazard being estimated as done in Section Computational considerations.
We also employ the *Sksurv* package for Python [26, 27], in particular the function *CoxnetSurvivalAnalysis* available in the imported submodule *sksurv.linear_model*. The function is called with parameter *l1_ratio* set to $0.99$ and *fit_baseline_model* set to *True*. In contrast to Tensorflow, the machine learning suite Sksurv actually allows one to compute full hazard and survival curves on a grid of x-values. We choose this option after fitting the model, and extract both curves with the help of the functions *predict_cumulative_hazard_function* and *predict_survival_function*, which return both curves as point sets.

For all approaches that make use of eq. (1), the baseline hazard $h_{0}(t)$ is fitted with the help of the *survival* R-package on CRAN [20]. Similarly, after fully specifying the hazard curve $h(t)$ of eq. (1), we always obtained the survival curve from the hazard curve via numerical integration as done in Section Numerical integration of the hazard curve to obtain thesurvival curve.

Importantly, since none of the aforementioned methods is designed for more than one input dataset, we apply all the above methods to the three datasets of Section Real datasets on AD by first concatenating them column-wise into one dataset, which then serves as the input to each method. This strategy of combining the three data layers a priori is equivalent to the *early fusion* approach of [14].

We evaluate all methods with respect to five metrics. Those are the normalized $L_{1}$ norm (normalized by the length of the vector), the AUC (area under the curve), the Concordance Index (C-Index) [28] and the Integrated Brier Score (IBS) [29, 30]. Moreover, we record the runtime of all methods. We display the mean of each metric across $100$ repetitions, together with a confidence band having coverage $1-\alpha $, where $\alpha =0.05$.

We compare the above approaches with our proposed cooperative polygenic hazard score model of Section Cooperativepolygenic hazard score models. The penalization parameter $\lambda $ in eq. (6) is determined for each application of the cooperative polygenic hazard score model via $10$-fold cross validation on the respective input data as described in [31, Section 7.10], where we employ the IBS as the performance metric. In $10$-fold cross validation, we divide the input data into $10$ parts, withhold each part one-by-one, and fit the parameter $\lambda $ to the other parts with the aim to minimize the cross-validation error given in [31, Eq. (7.49)]. The parameter $\rho $ of eq. (4) is chosen manually as $\rho =0.5$ (for three data layers, since in this case there are two penalties, one fusing the prediction of the first and the second data layer, and one fusing the prediction of the second and the third data layer), or $\rho =1$ (for two data layers) to equally weigh the data layers.

As discussed in [10], the *survival* R-package on CRAN [20] was not capable of fitting full hazard and survival models with the number of covariates we consider in the datasets of Section Real datasets on AD. We therefore omit it in the experiments of this section.

All computations were run on an Intel i5-7000U CPU processor with four cores, each having a 2.50 GHz clock speed, and 7.6 GiB RAM. The operating system was Kubuntu 22.04.

### Hazard and survival curves

We are interested in computing hazard and survival models based on the three data layers introduced in Section Real datasets on AD. In particular, we are interested in investigating in what way fusing the three data layers yields different polygenic hazard score models and predictions than computing the cooperative polygenic hazard score model of Section Cooperative polygenic hazard score models.

We look at two examples, first the Cox-lasso of [10] applied to the concatenation of the three data layers of Section Real datasets on AD, and second the proposed cooperative polygenic hazard score model of Section Cooperative polygenic hazardscore models, which draws separate inference from all three data layers (without combining them into one dataset). The Cox-lasso is chosen as it is the closest relative to the proposed approach of Section Cooperative polygenic hazard score models, and thus it is most suited to investigate the effect between using either a concatenated dataset or cooperative learning.

For the results presented in this section, we split the datasets of Section Real datasets on AD into a training set consisting of $300$ randomly selected individuals. The remaining unseen individuals are used as a validation set.

**Figure 3:**
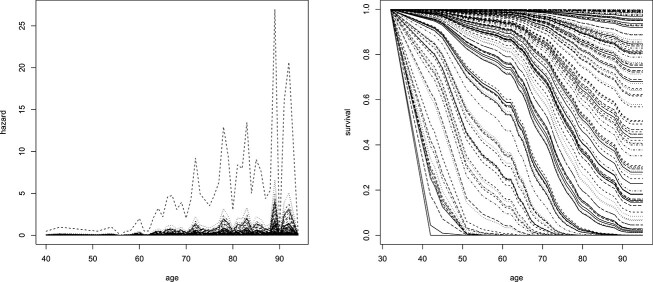
Real datasets of Section Real datasets on AD. Hazard curves (left) and survival (right) curves for the Cox-lasso of [10] are applied to the validation dataset. Each curve corresponds to one individual in the validation dataset.

Figure 3 shows the hazard and survival functions computed with the Cox-lasso on the concatenated datasets. Several observations are noteworthy. The estimated hazard is not monotonically increasing, as expected, but the hazard shows an overall increasing trend with age. The corresponding survival curves show a decreasing survival with age. As desired, we observe a wide spectrum of survival curves in Figure 3, ranging from individuals with low survival at ages as low as $40$ to individuals with high survival at age $90$ and beyond. However, the survival seems to be underestimated for some individuals and overestimated for others, as the observed number of individuals with a negligible survival at age $40$ seems too high, while for other individuals the survival is almost one even in the age bracket of $[90,100]$ years.

**Figure 4:**
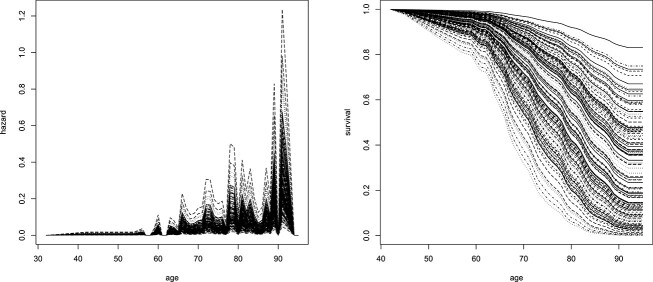
Real datasets of Section Real datasets on AD. Hazard curves (left) and survival (right) curves for the cooperative polygenic hazard score model of Section Cooperative polygenic hazard score models are applied to the validation dataset. Each curve corresponds to one individual in the validation dataset.

Figure 4 shows the results for the same data evaluated with the cooperative approach of Section Cooperative polygenic hazardscore models. In contrast to Figure 3, the hazard is estimated to be less extreme than in Figure 3, which can be seen by looking at the range of values on the y-axis of the hazard plot. Importantly, the corresponding survival curves in Figure 4 seem more meaningful, as they show a clear decrease in AD-free survival for all individuals until the age of around $90$, while still displaying a wide variety of functional shapes. In contrast to Figure 3, the predictions in Figure 4 avoid extreme cases such as individuals with low survival at age $40$ or those with survival of $1$ past the age of $100$.

### Comparison with other popular approaches

We adapt the experimental setting of [10] to evaluate the quality of the estimated hazard and survival curves with all the approaches introduced in Section Experimental setup. This experimental setting is as follows.

We are interested in the behavior and accuracy of all methods when scaling the size of the input data. To have a meaningful input, we resort to bootstrapping the datasets of Section Real datasets on AD. However, in order to be able to make meaningful predictions even for the boostrapped datasets, one has to keep the data layers separate for cases and controls. In particular, for any desired size $s \in{\mathbb{N}}$ (the number of bootstrapped individuals in the generated data), we first sample with replacement a new vector of indicators for cases and controls. For all resampled cases, we draw their age, APOE status, principal components, genetic data and cpgs data independently, entry by entry and with replacement, from the pool of cases. For controls, we proceed similarly using only the pool of controls. In order to vary the difficulty of the prediction, among other techniques, both the groups of cases and controls can be artificially stratified further by tuning either the probabilities for the resampled APOE status, or the age (age of last follow-up for controls, and age of onset for cases) between both groups. We decided to stratify the age distributions by artificially separating them by $5$ years. The added stratification also serves the purpose of overcoming the drawing of matched datasets.

Using this bootstrapping technique, we are able to generate three meaningful datasets for any new number of bootstrapped individuals. In the following experiments, we vary the size from $s=1000$ to $s=10\,000$ in steps of $1000$. Each new dataset is split randomly into a training set of size $0.5s$ and validation set of size $0.5s$. All the methods we evaluate are trained on the same training set and evaluated on the same validation set.

We evaluate the accuracy of the estimated hazard and survival curves as follows. For all individuals in the validation set, we know both their AD status and age. Here, the age is the age at the most recent follow-up for AD controls, and the age of onset for AD cases. Given the survival curve for an individual in the validation set, we look at the predicted survival at the age given in their record. For any control in the validation set, we would expect a higher predicted survival, while for cases the survival is expected to be lower. We then compare the known true binary AD outcome ($0$ for controls and $1$ for cases) with the predicted survival using the metrics introduced in Section Experimental setup, precisely the normalized $L_{1}$ loss (lower bounded by $0$ with $0$ being the best), the AUC (ranging from $0$ to $1$ with $1$ being the best), the Concordance Index (ranging from $0$ to $1$ with $1$ being the best) and the IBS (lower bounded by $0$ with $0$ being the best). Since the size of the datasets $s$ is varied, we divide the $L_{1}$ loss by the size of the validation set to make the metric comparable across datasets of different sizes.

**Figure 5:**
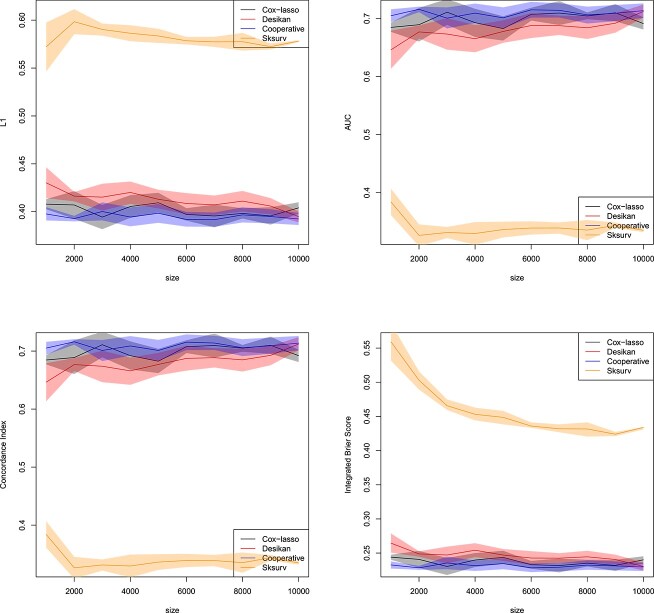
Real datasets of Section Real datasets on AD. Normalized $L_{1}$ loss between predicted survival and true AD status (top left), AUC (top right), Concordance Index (bottom left) and IBS (bottom right) for all individuals in the validation set as a function of the size of the training set. Confidence bands with coverage $1-\alpha $ are depicted around each mean in the same color, where $\alpha =0.05$. All the methods of Section Experimental setup are included in the comparison. Plotted with jittering.

Figure 5 displays all four metrics to assess the predicted survival and the true AD status. We observe that Sksurv incurs the highest $L_{1}$ error, while all other methods perform comparably in terms of $L_{1}$ loss. A similar picture is obtained when looking at the AUC, the Concordance Index or the IBS. Our proposed cooperative polygenic hazard score model attains the lowest average $L_{1}$ loss and, in a similar fashion, the highest average AUC and average Concorance Index, and lowest average IBS, though only by a slightly margin. The attained AUC is in the vicinity of $0.7$ and thus reasonable for AD prediction.

**Figure 6:**
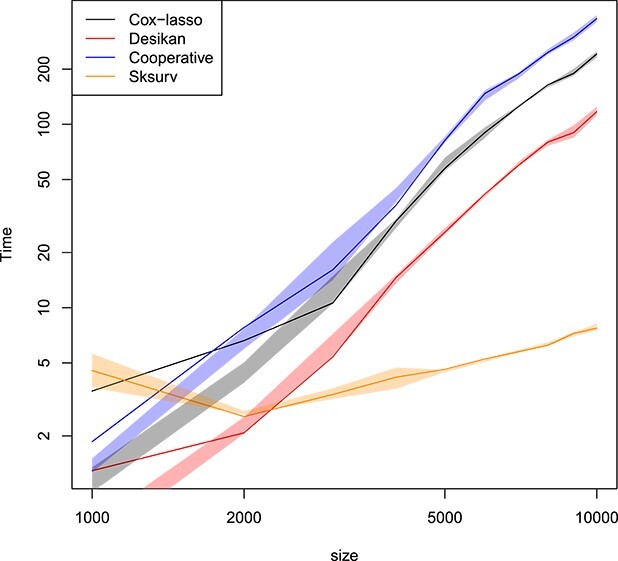
Real datasets of Section Real datasets on AD. Runtime in seconds for all methods of Section Experimental setup. Confidence bands with coverage $1-\alpha $ are depicted around each mean in the same color, where $\alpha =0.05$. Log scale on both axes. The empirical slope estimates are roughly $0.4$ for Sksurv, and $2.1$ for all other methods. Plotted with jittering.

Lastly, we look at the computational effort of all methods. For this, we time the computations of the hazard and survival curves reported in Figure 5. We display the runtimes in Figure 6 using a log–log plot to visualize asymptotic scalings. We observe that the Cox-lasso, the approach of [9], and the cooperative polygenic hazard score model roughly attain equal runtime scalings. An empirical line fit results in a slope of roughly $2.3$ for those methods, thus indicating a quadratic scaling of the computational runtime. The Sksurv machine learning approach has a lower (faster) asymptotic scaling (with a slope estimated to be roughly $0.4$).

### Simulated genetic and methylation datasets

We repeat the experiment of Section Comparison with otherpopular approaches using simulated data, precisely two data layers consisting of genetic information and methylation data for a simulated AD response.

The datasets are generated as follows. Let $n$ be the number of simulated individuals, $n_{g}$ be the number of desired genetic loci and $n_{m}$ be the number of desired methylation CpG sites. Moreover, let $n_{p} < \min (n_{g},n_{m})$ be the number of pairs of genetic loci and methylation CpG sites which are correlated.

For each genetic locus to be simulated, we generate genetic information for all subjects by drawing $n$ times from a binomial distribution of size $2$ and some probability *maf* (the minor allele frequency of the locus). We repeat this independently for each genetic locus, where the minor allele frequency *maf* of each locus is itself drawn from a Balding–Nichols model [32]. The Balding–Nichols model offers a statistical description of allele frequencies and assumes that they can be described by independent draws from a $Beta(p(1-F)/F,(1-p)(1-F)/F)$ distribution, where $p$ is the background allele frequency and $F$ is Wright’s fixation index $F_{ST}$ describing the population differentiation. We employ the parameter $F=0.001$ and draw $p$ from a uniform distribution in $[0,0.1]$.

The above procedure results in a vector $v_{g} \in{\mathbb{R}}^{n}$ of simulated genetic information for each locus. To simulate a vector of methylation CpG sites, we set $v_{m}=b \cdot v_{g} + \epsilon \in{\mathbb{R}}^{n}$, where $b=\sqrt{h^{2}/(2 maf (1-maf))}$ with $h=0.3$, and where the entries of $\epsilon \in{\mathbb{R}}^{n}$ are drawn from a Normal distribution with mean $0$ and standard deviation $\sqrt{1-h^{2}}$. In order to generate $n_{p}$ correlated pairs, we repeat the above generation mechanism and filter for those pairs satisfying $cor(v_{g},v_{m}) \in [0,0.1]$. The remaining $n_{g}-n_{p}$ genetic loci are generated as described previously, and likewise the remaining $n_{m}-n_{p}$ methylation CpG sites are drawn from a Normal distribution with mean $0$ and standard deviation $\sqrt{1-h^{2}}$. We can summarize the $n_{g}$ simulated genetic loci in a matrix $X_{g} \in{\mathbb{R}}^{n \times n_{g}}$, where each locus corresponds to a column, and likewise we summarize the $n_{m}$ methylation CpG sites in a matrix $X_{m} \in{\mathbb{R}}^{n \times n_{m}}$.

Last, to generate a response, we multiply the concatenated matrix $X=[X_{g},X_{m}]$ (concatenated by columns) with a random vector $v$ of length $n_{g}+n_{m}$, where each entry is independently drawn from a uniform distribution in $[0,1]$. The resulting vector $Xv$ is scaled into the interval $[50,100]$ and used as the age variable. To simulate an AD status, we uniformly draw an age of onset for each of the $n$ individuals in the interval $[50,100]$. If the age of onset is less than the simulated age variable, the response (the AD status) is set to $1$ for that particular subject, and $0$ otherwise.

We employ the aforementioned data generation mechanism to generate a dataset for $n=100$ individuals, each with $n_{g}=100$ genetic loci and $n_{m}=100$ methylation CpG sites. We vary the number $n_{p}$ of correlated pairs from $10$ to $90$ in steps of $10$, thus resulting in nine datasets, and evaluate all models of Section Experimental setup on the simulated dataets. Each time, we use a proportion of $0.7$ for training, and the remaining $0.3$ for validation.

**Figure 7:**
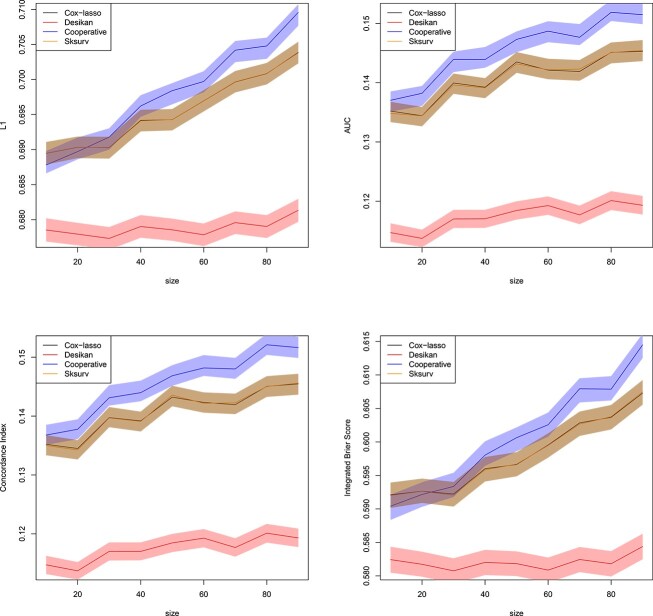
Simulated datasets of Section Simulated genetic and methylation datasets. Normalized $L_{1}$ loss between predicted survival and true AD status (top left), AUC (top right), Concordance Index (bottom left) and IBS (bottom right) for all individuals in the validation set as a function of the size of the training set. Confidence bands with coverage $1-\alpha $ are depicted around each mean in the same color, where $\alpha =0.05$. All the methods of Section Experimental setup are included in the comparison. Plotted with jittering.

Figure 7 shows the results of this simulation experiment as a function of the number $n_{p} \in \{10,20,\ldots ,90\}$ of correlated loci, evaluated again using the normalized $L_{1}$ loss, the AUC, the Concordance Index and the IBS. We observe that the prediction quality is lower than in the real data experiment, an artifact which is attributed to the data generation mechanism. However, the proposed cooperative polygenic hazard score model performs competitively with respect to the other methods. It achieves the best AUC and Concordance Index, while the method of [9] achieves the lowest $L_{1}$ norm and the lowest IBS.

### Ablation study

We are interested in assessing the performance of the cooperative polygenic hazard score model on the agreement parameter (that is, the agreement penalty) $\rho $, see eq. (6). The purpose of the agreement parameter $\rho $ is to enforce the consistency of the predictions made with the different data layers (see Section Incorporation of several data layers via cooperative learning).

We employ the same simulation setting as in Section Simulated genetic and methylation datasets, for which results were already presented in Figure 7. This setting consists of $m=2$ data layers, therefore eq. (6) can be explicitly written as 


(9) \begin{align*} \min_{\beta_{1},\beta_{2}} \left[ -\frac{1}{n} \sum_{i=1}^{2} l_{Z_{i}}(\beta_{i}) + \frac{\rho}{2} \left( Z_{1} \beta_{1} - Z_{2} \beta_{2} \right)^{2} + \lambda \sum_{i=1}^{2} \left| \frac{\beta_{i}}{\hat{\beta}_{i}} \right| \right],\end{align*}

where $\beta _{1}$ is the parameter vector for the fit of the first data layer $Z_{1}=X_{g}$ (genomic data), $\beta _{2}$ is the corresponding vector for the second data layer $Z_{2}=X_{m}$ (methylation data), $l_{Z}(\beta )$ is the likelihood function given in eq. (2), $\lambda $ is the lasso-type $L_{1}$ penalty and $\hat{\beta }_{1}$ and $\hat{\beta }_{2}$ are the maximizers of the partial log likelihood of eq. (2), respectively. The data layers $Z_{1} \in{\mathbb{R}}^{100 \times 100}$ (simulated genetic data with $n_{g}=100$ genetic loci) and $Z_{2} \in{\mathbb{R}}^{100 \times 100}$ (simulated methylation data with $n_{m}=100$ methylation CpG sites) are the same as the ones described in Section Simulated genetic and methylation datasets for $n=100$ individuals. We are interested in the behavior of the proposed cooperative polygenic hazard score model on the agreement parameter $\rho $ which we vary in $\rho \in \{0,0.5,1,2\}$. The lasso-type $L_{1}$ penalty $\lambda $ is fitted with cross-validation as in the previous experiments.

**Figure 8:**
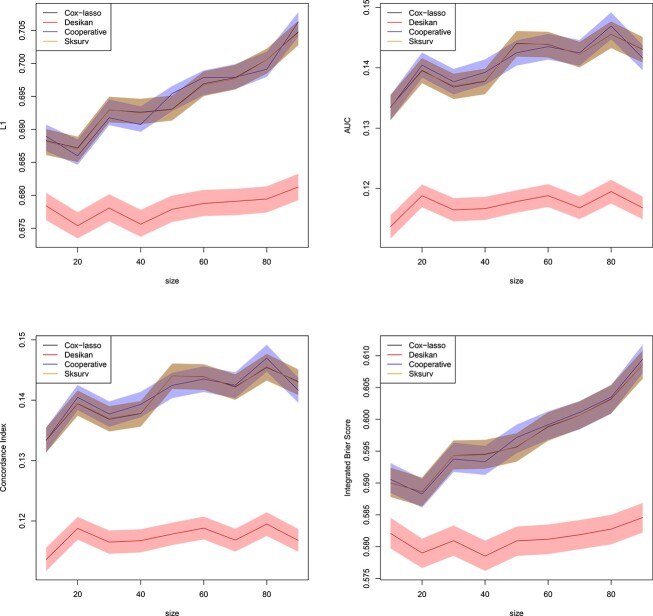
Ablation study with $\rho =0$ using the simulated datasets of Section Simulated genetic and methylation datasets. Normalized $L_{1}$ loss between predicted survival and true AD status (top left), AUC (top right), Concordance Index (bottom left) and IBS (bottom right) for all individuals in the validation set as a function of the size of the training set. Confidence bands with coverage $1-\alpha $ are depicted around each mean in the same color, where $\alpha =0.1$. All the methods of Section Experimental setup are included in the comparison. Plotted with jittering.

**Figure 9:**
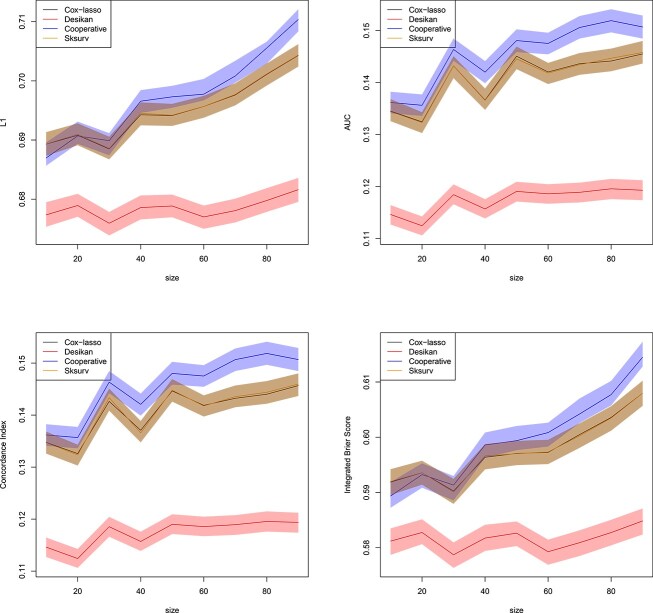
Ablation study with $\rho =0.5$ using the simulated datasets of Section Simulated genetic and methylation datasets. Normalized $L_{1}$ loss between predicted survival and true AD status (top left), AUC (top right), Concordance Index (bottom left) and IBS (bottom right) for all individuals in the validation set as a function of the size of the training set. Confidence bands with coverage $1-\alpha $ are depicted around each mean in the same color, where $\alpha =0.1$. All the methods of Section Experimental setup are included in the comparison. Plotted with jittering.

**Figure 10:**
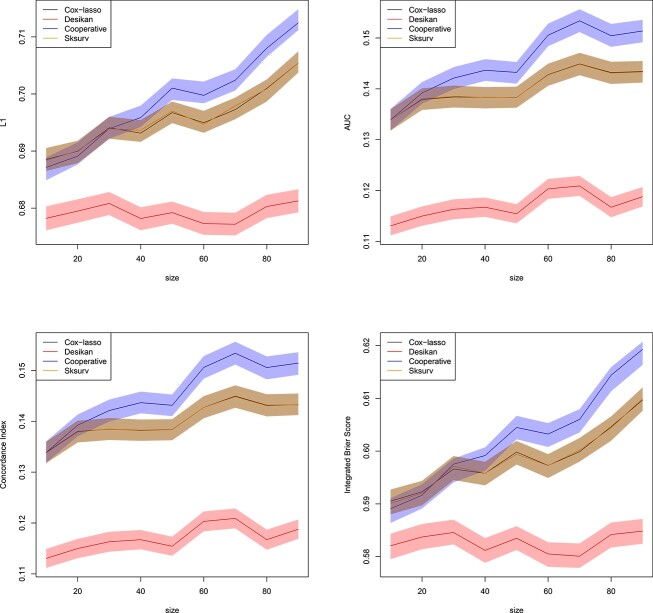
Ablation study with $\rho =2$ using the simulated datasets of Section Simulated genetic and methylation datasets. Normalized $L_{1}$ loss between predicted survival and true AD status (top left), AUC (top right), Concordance Index (bottom left) and IBS (bottom right) for all individuals in the validation set as a function of the size of the training set. Confidence bands with coverage $1-\alpha $ are depicted around each mean in the same color, where $\alpha =0.1$. All the methods of Section Experimental setup are included in the comparison. Plotted with jittering.

Figure 8 shows the results of this experiment for $\rho =0$. We observe that when the agreement of the predictions is not enforced in the cooperative polygenic hazard score model, the advantage in AUC and Concordance Index which we observed in Figure 7 is lost, and the proposed cooperative polygenic hazard score model performs similarly to the other approaches. Here, the results in Figure 7 are used as a baseline, which were computed with $\rho =1$ (see Section Experimental setup). Importantly, in the case $\rho =0$, solely the agreement of the predictions is not enforced; the two data layers are still used for prediction as they still both count toward the likelihood functions $l_{Z_{1}}(\beta _{1})$ and $l_{Z_{2}}(\beta _{2})$, see eq. (9). Increasing the agreement parameter to $\rho =0.5$ in Figure 9 reveals an increase in performance of the cooperative polygenic hazard score model, which is reflected, for instance, in the observed increase in the AUC or C-Index metrics. The increase in performance becomes even more pronounced with a further increase of $\rho $ to $\rho =2$ in Figure 10. Although not shown here, as $\rho \rightarrow \infty $, the model will eventually only emphasize the agreement of the predictions and neglect the fit to the response $y$, in which case the prediction accuracy will decrease again.

## Discussion

In this contribution, we propose a cooperative polygenic hazard score model that allows one to predict the disease-free survival of a patient for a specific trait or disease by integrating multiple layers of genetic (or other) information. Importantly, the proposed methodology combines the advantages of computing a time dependent prediction of the risk (or survival) for a disease with the ability to draw inference from several data layers. To this end, the proposed approach fuses two recent methodological advances, the one of [10] to compute time-dependent polygenic hazard score models, and cooperative learning of [14].

The proposed methodology provides a practitioner with a more differentiated view of the absolute risk of contracting a disease than the one given by merely a point estimate (for instance, a polygenic risk score). Using cooperative learning of [14], we can draw valid and coherent inference from several data layers, (potentially) resulting in a greater predictive power for the outcome of interest than predictions that are based on a single platform only.

To showcase our methodology, we compare our approach in the context of AD. By benchmarking against state-of-the-art approaches, such as the one of [9] as well as machine learning methods, we demonstrate that cooperative learning can help to make more accurate predictions of the disease-free survival. In the experiments using real AD data, the cooperative polygenic hazard score model achieves a slight edge over other state-of-the-art approaches. However, this improvement is incremental, which can be explained by the fact that an AUC of above $0.7$ is already in the vicinity of the best-known AUC values achieved for the AD prediction task, and that the noise in the real data we employ might not admit for more accurate predictions. In turn, in the experiments using simulated data layers, the proposed methodology shows a clear improvement in AUC and Concordance Index over the other approaches.

Additionally, the main advantage of the proposed cooperative polygenic hazard models (in comparison with black-box machine learning models) is their interpretability. The obtained models yield state-of-the-art or better performance in our experiments while providing (1) a linear score that can be easily interpreted, and (2) a weighting of the predictive power of the involved data layers. In particular, a weighting of the predictive power of the involved data layers is currently not returned by any other prediction model.

The proposed methodology is phrased in a general way, in the sense that it applies to any model that aims to fit a polygenic hazard score model using $m \in{\mathbb{N}}$ arbitrary data layers on $n$ individuals to a response $y \in{\mathbb{R}}^{n}$, which can be binary or continuous. Apart from the assumption that the censoring and event times are independent, we do not impose any other constraints on the event times, the binary censoring indicators, the data layers or the response (see Section Methods). This allows for a broad applicability of the proposed methodology to any arbitrary disease, as long as independent censoring and event times and certain data layers with predictive power are available. The performance of our proposed methodology will depend on the predictive power of the employed data layers. One crucial aspect here is the scaling of the proposed methodology. Indeed, empirical runtimes indicate a quadratic scaling for computing our cooperative polygenic hazard score models (see Section Comparison with other popular approaches). This will allow for the fitting of models with several thousand or ten thousand individuals. However, further methodological advances including the use of parallel computing will be required to scale the methodology to even larger datasets. Nevertheless, once a model has been fitted, the obtained coefficients $\beta _{1},\ldots ,\beta _{m}$ (see Figure 1) can be used to predict the hazard and survival as a function of age for a new individual instantly, as this only requires evaluating eq. (5) on the characteristics of the new individual for which a forecast is sought.

One potential limitation that is noteworthy in our AD application is the nature of the matched data we considered. Since the cases and controls in our data were matched by APOE, sex and age, it was expected that there is little stratification among cases and controls in the analysis, which can result in an AUC of around $0.5$. We aimed to overcome this drawback, which is introduced through matching, by artificially separating the two groups again by age when generating data layers of various sizes via bootstrapping.

**Key Points**

- We propose new methodology to combine both cooperative learning, a recent approach to leverage the predictive power of several datasets, and polygenic hazard score models. Polygenic hazard score models provide a practitioner with a much more differentiated view of the predicted disease-free survival than the one given by merely a point estimate, for instance computed with a polygenic risk score.
- We demonstrate that the prediction of the disease-free survival (in our case, of Alzheimer’s disease) can be improved by leveraging the predictive power of several datasets via cooperative learning for polygenic hazard score models.
- The proposed methodology returns (1) a linear score that can be easily interpreted (in contrast to machine learning approaches), (2) a weighting of the predictive power of the involved data layers and (3) individual survival curves for each patient.

## Author Biographies

**Georg Hahn** is a faculty member in the Division of Pharmacoepidemiology and Pharmacoeconomics of the Department of Medicine at Brigham and Women’s Hospital and Harvard Medical School, and an instructor in the Department of Biostatistics of the Harvard T.H. Chan School of Public Health. He holds an MSt in mathematics from Cambridge University and a PhD in statistics from Imperial College London.

**Dmitry Prokopenko** is an instructor at the Genetics and Aging Unit and McCance Center for Brain Health, Massachusetts General Hospital and Harvard Medical School. His research interests focus on Alzheimer’s disease genetics and statistical and computational problems posed by omics data.

**Julian Hecker** is an investigator in the Channing Division of Network Medicine at Brigham and Women’s Hospital and instructor in Medicine at Harvard Medical School. He holds a BSc in Mathematics, a BSc in Economics, a Master’s degree in Mathematics, and a PhD in Epidemiology.

**Sharon Lutz** is an assistant professor in the Department of Population Medicine at Harvard Medical School and Harvard Pilgrim Health Care Institute. She holds a BA and MA in Mathematics and a MA and PhD in Biostatistics.

**Kristina Mullin** is a senior laboratory manager and researcher in the Genetics and Aging Research Unit at The Massachusetts General Hospital. Her research interests are in genetic factors leading to Alzheimer’s disease. She holds a BSc in Biology.

**Leinal Sejour** is an analyst in the Bioinformatics Unit of the Department of Pathology of Beth Israel Deaconess Medical Center.

**Winston Hide** is an associate professor at Harvard Medical School and Beth Israel Deaconess Medical Center (BIDMC). He is also a co-Director of the Non-Coding RNA Precision Diagnostics and Therapeutics Core Facility. His group develops and performs data-driven systems approaches to disease causal discovery.

**Ioannis Vlachos** is an assistant professor of Pathology in the Harvard Medical School and the Director of the Bioinformatics Unit of the Non-coding RNA Precision Diagnostics and Bioinformatics Core.

**Stacia DeSantis** is a professor of Biostatistics at the University of Texas Health Science Center at Houston (UTHealth).

**Rudolph Tanzi** is the director of the Genetics and Aging Research Unit, Co-Director of the McCance Center for Brain Health, Co-Director of the Mass General Institute for Neurodegenerative Disease, and Vice-Chair of Neurology (Research) at Massachusetts General Hospital, and the Joseph P. and Rose F. Kennedy Professor of Neurology at Harvard Medical School. His main research focus is on Alzheimer’s disease, its genetics, treatment and prevention. Dr. Tanzi co-discovered the first three Alzheimer’s disease genes, including APP and directs the Cure Alzheimer’s Fund Alzheimer’s Genome Project, which identified the first neuroinflammation-related Alzheimer’s gene, CD33.

**Christoph Lange** is a professor of Biostatistics at the Harvard T.H. Chan School of Public Health. His research interests are at the intersection of biostatistical methodology, numerical analysis and computer science.
